# Impaired Grid-Like Representations at Theta Frequency in Schizophrenia

**DOI:** 10.1192/bjo.2022.185

**Published:** 2022-06-20

**Authors:** Laura Convertino, Daniel Bush, Fanfan Zheng, Rick Adams, Neil Burgess

**Affiliations:** 1 UCL, Institute of Cognitive Neuroscience, London, United Kingdom; 2 UCL, Institute of Neurology, London, United Kingdom; 3 Brainnetome Center, Beijing, China; 4 UCL, Centre for Medical Image Computing, London, United Kingdom; 5 UCL, Wellcome Centre for Human Neuroimaging, London, United Kingdom

## Abstract

**Aims:**

Schizophrenia is a chronic brain disorder characterised by distortion of thoughts and perception. Several studies have shown a key role of the hippocampal formation in the pathophysiology of schizophrenia. Patients show impaired theta coherence between medial temporal lobe and medial prefrontal cortex (mPFC), and impairment of knowledge structuring and inferential processes. Both the hippocampal formation and mPFC contain hexadirectional modulation of activity, indicative of grid cell populations. Grid cells play an important role in mapping the environment and are believed to represent the transition structure between task states. With other cell populations in the hippocampal formation, they play a fundamental role in inference, episodic memory, and spatial navigation. Here, we investigate whether schizophrenia is associated with disrupted grid firing patterns.

**Methods:**

To test this hypothesis, we asked 18 participants with diagnoses of schizophrenia and 26 controls (matched for age, sex and IQ) to perform a spatial memory task in magnetoencephalography (MEG), while navigating a virtual reality environment. We first analysed theta (4–10 Hz) power during movement onset compared to stationary periods. We then source-localised the signal and looked for the hexadirectional modulation of theta band oscillatory activity by heading direction during movement onset. We also controlled for other symmetries in theta frequencies (four, five, and eight fold) and hexadirectional modulation in other frequencies. The same participants performed an inference task outside MEG, which we used for correlation analysis.

**Results:**

The peak of theta power during movement onset was stronger in controls compared to patients (p < 0.05). In the control group, we found hexadirectional modulation of theta power by movement direction in the right entorhinal cortex (p < 0.005). This effect was absent in patients with a significant difference between groups (p < 0.05), suggesting that their entorhinal grid firing patterns may be disrupted. No other symmetry modulated theta power significantly in controls or patients, and hexadirectional modulation during movement onset was found only in theta frequencies in controls. Performance in the inference task was significantly impaired in schizophrenic patients, and spatial memory performance in both controls and patients was positively correlated with their performance in the inference task.

**Conclusion:**

These results are consistent with the hypothesis that impairments in knowledge structuring and inference associated with schizophrenia may arise from disrupted grid firing patterns in entorhinal cortex. Although further work is needed to better understand the role of grid cells in health and disease, this work provides new insights into dysfunction of the hippocampal formation in schizophrenia.

